# Chemical Composition and Antioxidant Characteristic of Traditional and Industrial Zhenjiang Aromatic Vinegars during the Aging Process

**DOI:** 10.3390/molecules23112949

**Published:** 2018-11-12

**Authors:** Chaoya Zhao, Ting Xia, Peng Du, Wenhui Duan, Bo Zhang, Jin Zhang, Shenghu Zhu, Yu Zheng, Min Wang, Yongjian Yu

**Affiliations:** 1 State Key Laboratory of Food Nutrition and Safety, Key Laboratory of Industrial Fermentation Microbiology, Ministry of Education, Tianjin Engineering Research Center of Microbial Metabolism and Fermentation Process Control, College of Biotechnology, Tianjin University of Science and Technology, Tianjin 300457, China; zcysuccess@foxmail.com (C.Z.); xiatingsyu@foxmail.com (T.X.); dp9298@163.com (P.D.); duanwhyy@163.com (W.D.); bozhango@163.com (B.Z.); ZHANGJIN1220@foxmail.com (J.Z.); yuzheng@tust.edu.cn (Y.Z.); 2 Jiangsu Hengshun Vinegar Industry Co., Ltd., Zhenjiang 212143, China; zshu72719@163.com

**Keywords:** Zhenjiang aromatic vinegar, aging process, proximate composition, organic acid, antioxidant activity, phenolic compounds

## Abstract

Zhenjiang aromatic vinegar (ZAV) is one of the well-known fermented condiments in China, which is produced by solid-state fermentation. It can be classified into traditional Zhenjiang aromatic vinegar (TZAV) and industrial Zhenjiang aromatic vinegar (IZAV) because of different production methods. The purpose of the study was to evaluate the variations and differences on chemical compositions and antioxidant activities of TZAV and IZAV during the aging process. The proximate composition, organic acids content, total phenolic content (TPC), total flavonoid content (TFC), total antioxidant activity (TAA) and phenolic compounds composition of TZAV and IZAV were detected during the aging process. Organic acids contents, TPC, TFC, TAA and phenolic compounds contents in ZAV were increased during the aging process. Acetic acid, lactic acid and pyroglutamic acid in ZAV were major organic acids. With the extension of aging time, TZAV and IZAV had similar proximate compositions and organic acids content. The values of TPC, TFC and TAA were higher in TZAV than in IZAV when aging is more than 3 years. Rutin and p-coumaric acid were detected in TZAV but not in IZAV. In principal component analysis (PCA), TZAV and IZAV can be divided into two groups according to their phenolic compounds composition. These findings provide references for evaluating TZAV and IZAV on the basis of their characterizations.

## 1. Introduction

Vinegar has been used as an acid condiment for thousands of years [1]. In European countries, liquid-state fermentation techniques are widely used for vinegar production. But in Asian countries, especially China and Japan, most vinegars are produced by typical solid-state fermentation techniques [2,3,4]. Based on the differences of raw materials, vinegars can be divided into grain vinegars and fruit vinegars. Chinese vinegar is produced from cereals and its manufacturing process mainly includes saccharification, alcohol fermentation, solid-state acetic acid fermentation, leaching, decoction and aging [5,6].

Zhenjiang aromatic vinegar (ZAV) is one of the famous Chinese vinegars, which is generally produced from glutinous rice, wheat bran, and rice hulls through solid-state fermentation [7,8]. ZAV is rich in organic acids, sugar, protein, amino acids and other nutrient and flavor ingredients, which largely affect its taste characteristics and organoleptic quality [9,10]. Furthermore, abundant antioxidant compounds, involving polyphenols, flavonoids, and melanoidins, were found in ZAV. These compounds play crucial roles in the prevention of diseases and contribute benefits to human health [11,12,13]. Flavor, nutrient and bioactive compounds in vinegars are mainly derived from raw materials and are accumulated through microbial metabolism during the production process. Complex chemical reactions and physical changes occur during the aging process, which can improve the taste and functional characteristics of vinegar [14,15,16,17].

**Figure 1:**
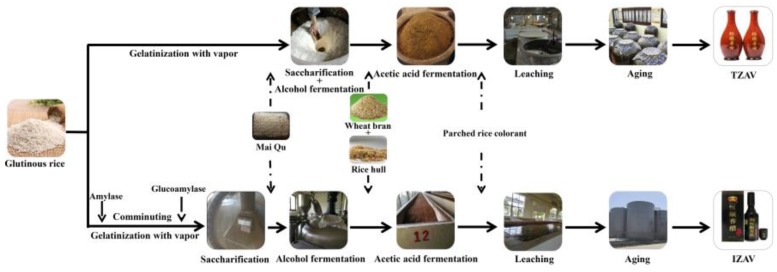
The production flow chart of traditional Zhenjiang aromatic vinegar (TZAV) and industrial Zhenjiang aromatic vinegar (IZAV).

There are mainly two vinegar manufacturing methods: the traditional method and the industrial method. The traditional method is a slow and complicated fermentation process, which are controlled empirically to facilitate microbial growth and accumulation of components in vinegar. The industrial method is a mechanical and fast production procedure. Advanced techniques and equipment are used in the industrial method, which can improve productivity and reduce production cost. Traditional Zhenjiang aromatic vinegar (TZAV) and industrial Zhenjiang aromatic vinegar (IZAV) are produced by these two production methods (Figure 1). With a mixture of cooked glutinous rice and unique Mai Qu in ceramic pots, starch saccharification and alcohol fermentation of TZAV are performed simultaneously. The acetic acid fermentation procedure requires experienced technicians turning over the Pei regularly. After leaching and decoction, TZAV is sealed and stored in ceramic pots on spacious ground for aging. In another manufacturing method, the raw materials of IZAV are crushed and cooked. Starch saccharification is accelerated by glucoamylase and then followed by alcohol fermentation in a large fermentation tank [18,19]. Subsequently, an automatic machine is used to turn over the Pei during acetic acid fermentation. Leaching and decoction are performed by equipment, and aging is conducted with mechanical devices in large jars [20]. Several authors have studied the characteristics of liquid-state fermentation vinegars, such as balsamic vinegar and grape wine vinegar, which are produced by two kinds of techniques (the traditional method and the industrial method). The two kinds of vinegars can be discriminated according to their chemical compositions and functional properties [21,22,23,24,25,26,27]. However, the differences in compositions and properties of solid-state fermentation vinegars, which were produced by traditional and industrial methods, have been rarely explored.

In this study, the proximate composition, organic acids content, total phenolic content, total flavonoids content, phenolic components and antioxidant activities of TZAV and IZAV were determined during the aging process. Principal component analysis (PCA) was performed on the composition of phenolic compounds to obtain information about the differences between TZAV and IZAV. The variations and differences were firstly evaluated on the chemical composition and antioxidant activities of TZAV and IZAV during the aging process. These results contribute to a theoretical basis for the effects of production techniques on the quality of ZAV, and provide references for the advancement of mechanical production procedures and manufacturing process standardization in ZAV.

## 2. Results and Discussion

### 2.1. Proximate Composition of Traditional Zhenjiang Aromatic Vinegar (TZAV) and Industrial Zhenjiang Aromatic Vinegar (IZAV)

The proximate composition of ZAV samples is summarized in Table 1. pH values in all ZAV samples varied from 3.38 to 3.62, which were in line with previous studies [28,29]. pH values were lower in IZAV samples than in TZAV samples. Total acidity is an important indicator for assessing the quality of vinegar [30]. All ZAV samples complied with regulatory limits for total acidity (Chinese National Standard: GB/T 18623-2011; total acidity ≥ 4.50 represent as qualified). There was no significant difference in the average values of total acidity between TZAV and IZAV samples (*p* > 0.05). Soluble solid content is the reflection of ingredients contained in liquid food, which has a significant influence on the taste of the food [31,32]. In this study, the content of soluble solid in ZAV samples ranged from 12.23 ± 0.00 to 23.02 ± 0.00 g/100 mL, which increased during 5 years of aging times. The average content of soluble solid were significantly higher in TZAV samples than in IZAV samples (*p* < 0.05), which is because longer fermentation time was possibly conducive to the accumulation of components in vinegar. Carbohydrates, fats and proteins are three main nutrients in foods, which provide energy sources for human body and constitute the structure and content of many cells [33,34]. Reducing sugars and amino nitrogen play an important role in the evaluation of quality in foods [35]. The content of protein in ZAV samples ranged from 0.86 ± 0.01 to 1.06 ± 0.03 g/100 mL. There was no significant difference between the average amount of protein in TZAV and those in IZAV (*p* > 0.05). The content of crude fat, amino nitrogen and carbohydrate in ZAV samples varied from 0.15 ± 0.03 to 0.44 ± 0.02 g/100 mL, from 0.21 ± 0.01 to 0.36 ± 0.00 g/100 mL and from 3.52 ± 0.12 to 5.36 ± 0.00 g/100 mL, respectively. The average amounts of amino nitrogen, crude fat and carbohydrate were significantly higher in TZAV samples than in IZAV samples (*p* < 0.05). The reducing sugar content of IZAV samples decreased gradually in 5 years of the aging process, which was in accordance with the finding of Wang et al. [36]. The average amount of reducing sugar was significantly higher in TZAV samples than in IZAV samples (*p* < 0.05). These findings suggest that the aging stage could enhance the quality of vinegar products. Even though IZAV samples had lower average content in many ingredients than TZAV samples, proximate compositions in IZAV samples are similar to those in TZAV samples with the extension of aging time.

### 2.2. Organic Acid Contents in TZAV and IZAV

Organic acids are important components of vinegar, which contribute to the specific flavor and organoleptic property of vinegars [37,38]. As shown in Table 2, the main organic acids of ZAV samples were detected by the high-performance liquid chromatography (HPLC) method. In ZAV samples, acetic acid, lactic acid and pyroglutamic acid were three major organic acids and represented more than 90% of the total organic acids. Acetic acid contents ranged from 1.58 ± 0.08 to 4.92 ± 0.17 g/100 mL, which mainly contributed to sourness in ZAV. The average content of acetic acid in TZAV samples was significantly higher than that in IZAV samples (*p* < 0.05). Lactic acid and pyroglutamic acid contents varied from 1.16 ± 0.05 to 3.41 ± 0.06 g/100 mL and from 0.22 ± 0.01 to 2.26 ± 0.17 g/100 mL, respectively, which increased with the extension of aging time. These values were consistent with values obtained by Xu et al., who reported the contents of acetic acid and lactic acid in ZAV [39]. In addition, TZAV-3 and TZAV-4 had higher succinic acid content than other samples. Malic acid, oxalic acid and tartaric acid contents varied from 0.01 ± 0.00 to 0.34 ± 0.01 g/100 mL, which presented lower levels in comparison to other organic acids. These results indicate that appropriate aging times could improve organic acid contents in ZAV samples, especially lactic acid and pyroglutamic acid, which belong to non-volatile acids. The accumulation of non-volatile acids could neutralize the pungent odor and harsh taste in ZAV.

### 2.3. Total Phenolic, Flavonoid Contents and Antioxidant Activities in TZAV and IZAV

Polyphenols and flavonoids are important bioactive compounds in foods, which have a significant contribution to defend against oxidative stress [40,41,42]. Table 3 shows total phenolic content (TPC) and total flavonoid content (TFC) in TZAV and IZAV. The lowest TPC and TFC were 2.07 ± 0.01 mg gallic acids equivalents (GAE)/mL and 1.21 ± 0.05 mg rutin equivalents (RE) /mL, respectively, which were determined in IZAV-1. The values of TPC and TFC in TZAV samples increased by 159.0% and 246.4% during 5 years of aging process, and TZAV-3 had the highest TPC and TFC (6.45 ± 0.19 mg GAE/mL and 5.44 ± 0.09 mg RE/mL). The values of TPC and TFC in IZAV samples increased by 96.6% and 47.9% during 6 years of aging process, and the highest TPC and TFC was determined in IZAV-8 (4.07 ± 0.11 mg GAE/mL and 3.00 ± 0.07 mg RE/mL). A tendency was obvious that values of TPC and TFC in TZAV and IZAV samples were increased with the extension of aging time, whereas these values decreased slowly in the later stage of the aging process. The average contents of TPC and TFC were higher in TZAV samples than in IZAV samples (TPC values: *p* < 0.05; TFC values: *p* > 0.05). There are differences in TPC and TFC between our results in ZAV and other results in varied vinegars due to different raw materials and production procedures. The TPC of red wine vinegars, which produced in barrels made from different woods, varied from 1006.19 to 1882.7 mg/L. The highest TPC of red wine vinegar was measured in Chestnut barrel [43]. In Shanxi aged vinegars, TPC and TFC contents measured 0.96 to 5.80 mg GAE/mL and 0.33 to 4.50 mg RE/mL, respectively, and increased with the extension of aging time [44]. TPC in cherry vinegars, which obtained by three varieties of cherry and two fermentation methods, ranged from 3.26 to 7.57 mg GAE/mL [45]. Verzelloni et al. [46] have shown that TPC and TFC ranged from 1.99 to 3.72 mg catechin/mL and from 2.29 to 3.34 mg catechin/mL in balsamic vinegars.

The antioxidant activities of vinegar mainly derived from its bioactive compounds [47,48,49]. Several studies have suggested that total antioxidant activity (TAA) of vinegars was highly correlated with TPC and TFC [50,51,52]. TAA in ZAV samples was investigated by 2,2-diphenyl-1-picrylhydrazyl (DPPH), ferric reducing antioxidant power (FRAP) and 2,2′-azino-bis(3-ethylbenzthiazoline-6-sulfonic acid) (ABTS) assays (Table 3). Values of DPPH, FRAP and ABTS in IZAV-1 were 17.82 ± 1.69, 9.25 ± 0.77 and 10.47 ± 1.02 mmol Trolox equivalent antioxidant capacity (TEAC)/L, respectively, which were lower than those in other samples. TAA values of TZAV and IZAV samples, which were measured by FRAP and ABTS assays, were similar at the beginning of aging processs. With the extension of aging time, the values of TAA were significant higher in TZAV samples than in IZAV samples except for DPPH values (*p* < 0.05). This difference could be due to the different reaction principles among DPPH, FRAP and ABTS assay [53,54]. In ZAV, the values of TAA were increased during 5 years of aging process, and then decreased slowly with the extension of aging time. This tendency was in line with TPC and TFC in ZAV samples, which indicated that the antioxidant power in vinegars was influenced by their TPC and TFC [55]. During the aging process, phenolic compounds interact with macromolecules, such as melanoidin, protein and polysaccharide, result in the formation of precipitates in vinegar. The decline in TPC, TFC and TAA of ZAV possibly due to precipitation of phenolic compounds in the later stage of the aging process [56,57]. The average antioxidant activity values of traditional and industrial Turkey grape vinegars (unaged) measured by ABTS assay were 13.50 and 10.37 mmol TEAC/L, respectively, which were consistent with our result [27]. The TAA of cherry vinegars was measured by Kawa-Rygielska et al. [45]. The result showed that TAA of coral-fruit cherry vinegar was 10.23 mmol TE/mL (DPPH method), and 3.6 mmol TE/mL (FRAP method), which were higher than other cornelian cherry vinegars. Moreover, the relationships between TAA of TZAV and IZAV and their TPC and TFC were shown in Table 4. In TZAV samples, the positive correlation was found between TAA and TPC evaluated by DPPH, FRAP and ABTS assays (r = 0.963, r = 0.998 and r = 0.987, respectively; *p* < 0.05). The TAA of IZAV was positively correlated with TFC (r = 0.985, r = 0.997 and r = 0.981, respectively; *p* < 0.05) when detected by DPPH, FRAP and ABTS assays. Similarly, TPC and TFC of IZAV samples were also significantly correlated with their TAA measured by DPPH, FRAP and ABTS assays. In general, these results indicate that TPC, TFC and TAA of ZAV were increased during the aging process, and TZAV samples were associated with higher values of TPC, TFC and TAA when the aging times more than 3 years. During the aging process, TPC and TFC have significant correlation with TAA in TZAV and IZAV samples.

### 2.4. Phenolic Compounds and Their Contributions to Total Antioxidant Activity (TAA) in TZAV and IZAV

**Figure 2:**
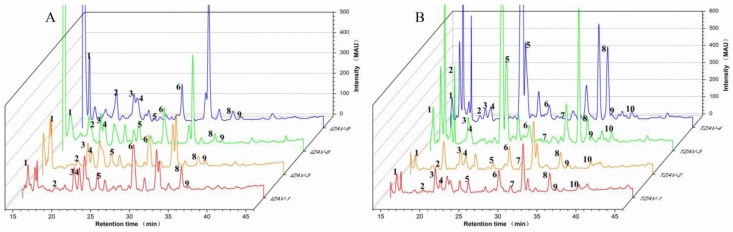
High-performance liquid chromatography (HPLC) chromatograms (recorded at 278 nm) of IZAV (**A**) and TZAV (**B**) samples showing the major phenolic compounds. Numbers represent different phenolic compounds (1: P-hydroxybenzoic acid; 2: chlorogenic acid; 3: caffeic acid; 4: vanillic acid; 5: syringic acid; 6: catechin; 7: p-coumaric acid; 8: ferulic acid; 9: sinapic acid; 10: rutin).

Ten phenolic compounds, including p-hydroxybenzoic acid, chlorogenic acid, caffeic acid, vanillic acid, syringic acid, catechin, p-coumaric acid, ferulic acid, sinapic acid and rutin were separated and quantified by the HPLC method (Figure 2). The distributions of phenolic compounds in extracts obtained from ZAV samples were presented in Table 5. Total phenolic contents in IZAV samples ranged from 84.36 ± 1.86 to 114.06 ± 3.57 mg/L, and those in TZAV samples ranged from 81.08 ± 3.94 to 133.92 ± 0.63 mg/L. Total phenolic contents by the HPLC method were significantly lower than those by Folin–Ciocalteu and colorimetric assays (*p* < 0.05), because more phenolic compounds were determined by Folin–Ciocalteu and colorimetric assays. In IZAV samples, rutin and p-coumaric acid were not detected or at lower levels than the limit of detection (LOD) of the method (0.04 and 0.02 μg/mL, respectively). Catechin in the ZAV samples contributed to 29.08–74.89% of total phenolic contents and decreased with the aging time. Catechin, syringic acid, p-hydroxybenzoic acid and chlorogenic acid were major phenolic compounds in TZAV samples. Catechin, vanillic acid, chlorogenic acid and caffeic acid were the main phenolic acids in IZAV samples. TZAV had higher average amounts of p-hydroxybenzoic acid and Syringic acid (7.46 ± 2.54 mg/L and 12.81 ± 11.37 mg/L) than IZAV (4.15 ± 1.75 mg/L and 3.08 ± 1.27 mg/L) (*p* < 0.05). The average contents of vanillic acid and catechin in IZAV (7.62 ± 2.07 mg/L and 64.17 ± 8.71 mg/L) was significantly higher than that in TZAV (5.76 ± 1.06 mg/L and 41.35 ± 5.56 mg/L) (*p* < 0.05). Abundant phenolic compounds also were detected in other vinegars. Bakir et al. [58] reported that apple vinegars contained abundant phenolic compounds, including gallic acid, p-hydroxybenzoic acid, catechin, syringic acid, p-coumaric acid and caffeic acid. Fourteen kinds of anthocyanin compounds were identified in strawberry vinegar, which pelargonidin 3-glucoside and pelargonidin 3-rutinoside were predominant phenolic compounds [59]. Yu et al. [60] found that ferulic acid, p-coumaric acid, protocatechuic acid, caffeic acid, vanillic acid and gallic acid were detected in oat and buckwheat vinegar, and contents of ferulic acid, p-coumaric acid, protocatechuic acid, caffeic acid and gallic acid were higher in oat vinegar than in buckwheat vinegar. Fruits and cereals are rich in phenolic compounds, which present in two forms: soluble-free and insoluble-bound. Soluble-free is a major form of phenolic compound in fruits, whereas a majority of phenolic compounds in cereals belong to the insoluble-bound form [61,62]. This is the reason why most of fruit vinegars possess more abundant phenolic compounds than cereal vinegars. Bound phenolics could be released by microbial catalysis during the production process of vinegar [63]. Thus, production techniques have great effects on phenolic compounds composition of vinegars.

**Figure 3:**
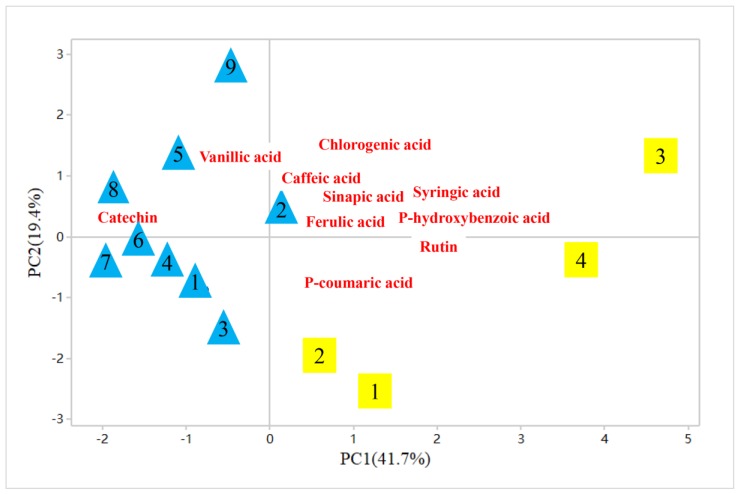
Principal component analysis (PCA) biplot displayed associations between phenolic compositions and two kinds of ZAV samples. Blue triangles represent IZAV and yellow squares represent TZAV.

Moreover, differences between TZAV and IZAV samples were evident from the PCA on compositions of phenolic compounds (Figure 3). Two principal components (PCs) expressed 61.1% of total variance. TZAV samples were located in the positive part of PC1, which showed the most discriminating variables 41.7% of the variance and positively associated with rutin, syringic acid, p-hydroxybenzoic acid and sinapic acid. IZAV samples were located in the negative part of PC1, which was associated with higher contents of vanillic acid and catechin. These results indicate that TZAV and IZAV samples can be divided according to different compositions and concentrations of phenolic compounds. The relationships between TAA of TZAV and IZAV samples and their main phenolic compounds contents are shown in Table 6. The results showed that syringic acid, sinapic acid and rutin contents of TZAV were positively correlated with TAA measured by DPPH, FRAP and ABTS assays (*p* < 0.05). In addition, DPPH values of TZAV were also positively correlated with their chlorogenic acid contents (r = 0.960, *p* < 0.05). A negative correlation was found between p-coumaric acid contents of TZAV samples and their ABTS values (r = −0.971, *p* < 0.05). In IZAV samples, caffeic acid contents were positively correlated with TAA measured by FRAP assays (*p* < 0.05). However, p-hydroxybenzoic acid contents had significantly negative correlation with TAA measured by DPPH, FRAP and ABTS assays (*p* < 0.05). Alonso et al. [64] analyzed the correlation between the TAA and phenolic compounds in sherry vinegars aged in wood and without wood. The results showed that TAA of sherry vinegars aged in wood was positively correlated with caffeic acid content whereas it was negatively correlated with ferulic acid contents. These results were opposite to the correlation between TAA of sherry vinegars aged without wood and their caffeic acid and ferulic acid contents. Xie et al. [44] reported the relationship between TAA and phenolic compounds in Shanxi aged vinegars during the brewing process. The results showed that TAA of Shanxi aged vinegars measured by ABTS and FRAP assays was positively correlated with their gallic acid, catechin and chlorogenic acid contents, whereas they were negatively correlated with their ferulic acid contents. These results indicate that phenolic compounds in TZAV and IZAV samples exert different contributions to TAA.

## 3. Materials and Methods

### 3.1. Samples and Reagents

Samples were collected by using sterilized cylinder-shaped samplers. In the Vinegar Culture Museum of China Zhenjiang (Jiangsu, China), TZAV was not collected at every aging year in ceramic aging pots. IZAV samples were collected from large glass aging jars in Jiangsu Hengshun Vinegar Industry Co., Ltd. (Jiangsu, China). TZAV1, TZAV-2, TZAV-3 and TZAV-4 represented samples with aging time of 0, 2, 5 and 7 years. IZAV1-9 represented samples with aging time of 0, 1, 2, 3, 4, 4.5, 5, 6 and 7 years. Every kind of vinegar was collected from three pots or jars at the same aging time. In total, 13 kinds of samples of ZAV were analyzed.

HPLC standards of organic acids and phenolic compounds were purchased from Sigma Aldrich (Deisenhofen, Germany). Folin–Ciocalteu reagent, DPPH, gallic acid and rutin were obtained from Sinopharm Chemical Reagent Co., Ltd. (Shanghai, China). Total antioxidant capacity assay kits with ABTS and FRAP were purchased from the Beyotime Institute of Biotechnology (Shanghai, China). 

### 3.2. Proximate Compositions

pH in ZAV samples was measured with a pH meter (Metrohm, Herisau, Switzerland). Total acidity of ZAV samples was determined by methods of the Chinese National Standard (GB/T 12456-2008). The protein content in ZAV was calculated by the Bradford method [65]. Contents of soluble solid, crude fat, reducing sugar and amino nitrogen in ZAV were determined according to the methods of the Chinese National Standard (GB/T 18187-2000; GB/T 5009.6-2016; GB/T 5009.7-2016; GB/T 5009.235-2016). Carbohydrate in ZAV sample was converted into reducing sugar after acid hydrolysis: 5 mL of sample was mixed with 5mL of HCl (21%, *w*/*v*). After 15 min at 70 °C, the mixture was neutralized with NaOH solution. The treated sample was determined by GB 5009.7-2016.

### 3.3. Analysis of Organic Acids

The contents of organic acids in ZAV samples were analyzed by an Agilent 1260 HPLC system (Agilent Corp., Karlsruhe, Germany); 2 mL of diluted samples was centrifuged at 6000 *g* for 10 min, and then the supernatant was filtered through 0.45 μm membrane. The HPLC working parameters were as follows: the chromatography column (Aminex HPX-87H lon Exculsion Column, 7.8 × 300 mm i.d., 5 μm), 0.6 mL/min of flow rate, 30 °C of column temperature, 20 μL of injection volume, the H_2_O containing 0.049% H_2_SO_4_ (*w*/*v*) as the mobile phase. Detection was performed by measuring the ultraviolet (UV) absorption at 215 nm. Identification of organic acids was conducted by comparing retention times and ultraviolet-visible (UV-Vis) data of 7 kinds of organic acid standards. A standard curve was obtained according to 8 concentration levels of organic acid standard. Concentrations of organic acids were quantified by their corresponding standard curves. The coefficients of all organic acids in standard curves were higher than 0.999 (Table S1). The precision of the HPLC methods were tested by measuring samples at 3 concentration levels for 6 times. The relative standard deviations (RSD) did not exceed 5%. The accuracy of the HPLC methods were checked by measuring standard addition recovery of samples. The values of standard addition recovery in the organic acids method were ranged from 92.1% to 98.5%. In addition, LOD and limits of detection quantitation (LOQ) of organic acids ranged from 0.01 to 0.03 μg/mL and 0.03 to 0.12 μg/mL, respectively.

### 3.4. Measurement of Total Phenolic and Flavonoid Contents

The TPC of the ZAV samples was determined by the Folin-Ciocalteu method. Every sample was diluted 10 times with distilled water, and 0.2 mL of diluted sample was mixed with 0.8 mL of Folin–Ciocalteu reagent. After 3–5 min, 1.5 mL of 10% Na_2_CO_3_ (*w*/*v*) solution was mixed and then distilled water was added to obtain a final volume of 10 mL. The mixture was measured at 765 nm after 120 min in the dark. Gallic acid was used as a reference, and the results were expressed as mg GAE/mL.

The TFC of ZAV samples was measured through a colorimetric assay. Every sample was neutralized with NaOH solution (2%, *w*/*v*) and diluted 10 times with distilled water. 2 mL of diluted sample, 8 mL of distilled water and 1 mL of NaNO_2_ solution (5%, *w*/*v*) were mixed. After 6 min, 1 mL of Al(NO_3_)_3_ solution (5%, *w*/*v*) was added and stood for 6 min. Finally, 4 mL of NaOH solution (20%, *w*/*v*) was added and made up to 25 mL with distilled water. After 15 min, the absorbance was measured at 510 nm. Rutin was used as a reference, and TFC was expressed as mg RE/mL.

### 3.5. Determination of TAA

The antioxidant activities of ZAV samples were evaluated by the DPPH radical scavenging activity assay. Briefly, 20 μL of diluted samples was mixed sufficiently with 180 μL of DPPH working solution. The mixture was incubated at room temperature for 30 min in the dark. The absorbance was measured at 517 nm with a microplate reader. A calibration curve was prepared with different concentrations of Trolox solutions, and the results were expressed as mmol TEAC/L of vinegar.

The ABTS radical scavenging capacities of ZAV samples were conducted with a Total Antioxidant Capacity Assay Kit. The ABTS radical cation (ABTS+) was generated by mixing an aqueous solution of ABTS and oxidant. These reagents reacted in a ratio of 1:1 (*v*/*v*) respectively and were kept to react completely in the dark for 12 h, then diluted to an absorbance of 0.70 ± 0.05 at 414 nm with ethanol. 200 μL ABTS working solution and 10 μL diluted samples were mixed. After 2–6 min in the dark, the absorbance was measured at 414 nm in a microplate reader. Trolox was used as a standard compound. The TAC was expressed as mmol TEAC/L of vinegar.

The reducing abilities of ZAV samples were measured by a Total Antioxidant Capacity Assay Kit with the FRAP method. A working solution was freshly prepared by mixing diluted solution, TPTZ, and detection buffer solution at a ratio of 10:1:1 (*v*/*v*/*v*). 180 μL of FRAP working solution and 5 μL of diluted sample were mixed and then incubated at 37 °C for 5 min. The absorbance was recorded at 593 nm with the microplate reader. Trolox was used as a reference compound. The results were calculated as reported as mmol TEAC/L of vinegar.

### 3.6. Identification of Phenolic Compositions

Phenolic compositions of ZAV were identified by the HPLC method. Every sample (5 mL) was ultrasonically extracted with 20 mL ethyl acetate three times. The extracts were mixed and evaporated to dry using a rotary evaporation instrument (40 °C). Subsequently, the residue was dissolved in 600 μL of 50% methanol (*v*/*v*). The solution was filtered through 0.45 μm membrane and injected into the HPLC system (Agilent Technologies Inc., California, CA, USA). The Phenyl chromatography column (250 mm × 4.6 mm i.d., 5 μm) was obtained from Nano-Micro (Suzhou, China). The following gradient system was used with water/acetic acid (98:2, *v*/*v*, solvent A) and water/acetonitrile/acetic acid (73:25:2, *v*/*v*/*v*, solvent B): 0 min, 5% B; 40 min, 30% B; 45 min, 20% B; 50min, 100% B; 51 min 5% B, and then held for 5 min. The flow rate was 1.00 mL/min, column temperature was 40 °C. UV-vis detection wavelengths were 278 nm. Identification of the major phenolic compounds was conducted by comparing retention times and UV-Vis data of phenolic standards. In addition, phenolic compounds were quantified by their corresponding standard curves. The coefficients of all phenolic compounds in standard curves were higher than 0.999 (Table S2). The precision of the HPLC methods were tested by measuring samples at 3 concentration levels 6 times. The relative standard deviations (RSD) did not exceed 5%. The accuracy of the HPLC methods were checked by measuring standard addition recovery of samples. The values of standard addition recovery in phenolic compounds methods ranged from 86.5% to 96.8%. LOD and LOQ of phenolic compounds ranged from 0.01 to 0.05 μg/mL and 0.06 to 0.12 μg/mL, respectively.

### 3.7. Statistical Analysis

All determinations were performed in triplicate. Results were presented as mean ± standard deviation (S.D.). All statistical analyses were performed in SPSS 24.0 for Windows (SPSS Inc., Chicago, IL, USA). Differences among the groups were tested by one-way analysis of variance (ANOVA) with the Duncan multiple comparison test. The Student’s *t*-test was used to evaluate difference between mean values of all parameters in TZAV samples and those in IZAV samples. The related correlation analyses were performed with the Pearson’s correlation test. To assess divergence in different values between TZAV and IZAV samples, PCA were performed using Minitab 17.0 (IBM Inc., New York, NY, USA).

## 4. Conclusions

In this study, the chemical compositions and functional characterizations between TZAV and IZAV samples were analyzed during the aging process. With the extension of aging time, TZAV and IZAV samples had similar proximate compositions and organic acid content. Moreover, the TZAV samples represented more abundant phenolic compounds and stronger antioxidant capacity than the IZAV samples. PCA evaluated the difference between the TZAV and IZAV samples according to compositions and proportions of phenolic compounds. This study proved the production method to have a significant impact on phenolic compounds’ composition and antioxidant capacity in ZAV during the aging process. These results contributed to better comprehension of the compositional and antioxidant characteristics of TZAV and IZAV samples during the aging process. In future, these parameters can be monitored to control the quality of ZAV and improve industrial procedures.

## Figures and Tables

**Table 1:** Proximate compositions of TZAV and IZAV samples ^1,^^2^.

Samples	Aging Time (Year)	pH	Total Acidity (g/100 mL)	Soluble Solid (g/100 mL)	Protein (g/100 mL)	Crude Fat (g/100 mL)	Carbohydrate (g/100 mL)	Reducing Sugar (g/100 mL)	Amino Nitrogen (g/100 mL)
TZAV-1	0	3.59 ± 0.00 b	5.80 ± 0.00 l	17.81 ± 0.01 e	0.86 ± 0.01 i	0.31 ± 0.03 b	5.36 ± 0.00 a	2.70 ± 0.06 c	0.26 ± 0.01 f
TZAV-2	2	3.61 ± 0.01 a	6.82 ± 0.01 b	17.23 ± 0.00 g	0.90 ± 0.01 gh	0.33 ± 0.02 b	5.43 ± 0.07 a	2.47 ± 0.01 e	0.32 ± 0.00 c
TZAV-3	5	3.62 ± 0.00 a	6.64 ± 0.00 d	23.02 ± 0.00 a	1.02 ± 0.02 b	0.32 ± 0.00 b	5.24 ± 0.08 b	2.60 ± 0.03 d	0.34 ± 0.00 b
TZAV-4	7	3.56 ± 0.00 c	6.48 ± 0.01 f	19.42 ± 0.00 b	1.03 ± 0.02 b	0.44 ± 0.02 a	4.82 ± 0.04 c	2.89 ± 0.06 b	0.36 ± 0.00 a
IZAV-1	0	3.41 ± 0.01 g	4.75 ± 0.01 m	12.23 ± 0.00 l	0.90 ± 0.01 gh	0.22 ± 0.00 d	4.35 ± 0.03 d	1.35 ± 0.03 j	0.21 ± 0.01 h
IZAV-2	1	3.47 ± 0.00 e	6.13 ± 0.00 i	12.56 ± 0.00 k	0.95 ± 0.01 de	0.15 ± 0.01 e	4.35 ± 0.04 d	2.13 ± 0.02 f	0.26 ± 0.03 f
IZAV-3	2	3.49 ± 0.01 d	6.09 ± 0.02 j	15.79 ± 0.01 i	0.88 ± 0.01 hi	0.23 ± 0.01 d	3.52 ± 0.12 f	3.00 ± 0.03 a	0.27 ± 0.00 ef
IZAV-4	3	3.41 ± 0.02 g	5.95 ± 0.00 k	14.18 ± 0.01 j	0.89 ± 0.01 h	0.43 ± 0.03 a	5.16 ± 0.09 b	2.40 ± 0.10 e	0.24 ± 0.00 g
IZAV-5	4	3.45 ± 0.01 f	6.41 ± 0.00 g	18.42 ± 0.00 d	0.97 ± 0.01 cd	0.31 ± 0.02 b	4.38 ± 0.00 d	1.59 ± 0.08 h	0.28 ± 0.00 def
IZAV-6	4.5	3.41 ± 0.01 g	6.84 ± 0.00 a	17.14 ± 0.01 h	0.92 ± 0.01 fg	0.21 ± 0.00 d	3.64 ± 0.04 e	1.78 ± 0.05 g	0.29 ± 0.01 de
IZAV-7	5	3.49 ± 0.01 d	6.32 ± 0.01 h	17.37 ± 0.01 f	0.98 ± 0.01 c	0.27 ± 0.03 c	3.62 ± 0.06 ef	1.45 ± 0.06 i	0.27 ± 0.01 ef
IZAV-8	6	3.44 ± 0.01 f	6.69 ± 0.01 c	17.81 ± 0.01 e	1.06 ± 0.03 a	0.22 ± 0.01 d	4.74 ± 0.10 c	2.67 ± 0.10 cd	0.30 ± 0.02 d
IZAV-9	7	3.38 ± 0.01 h	6.61 ± 0.00 e	18.79 ± 0.00 c	0.93 ± 0.01 ef	0.43 ± 0.00 a	5.37 ± 0.07 a	2.72 ± 0.03 c	0.29 ± 0.03 de
Mean values									
TZAV		3.60 ± 0.26 A	6.44 ± 0.40 A	19.37 ± 2.36 A	0.95 ± 0.08 A	0.35 ± 0.06 A	5.21 ± 0.25 A	2.67 ± 0.16 A	0.32 ± 0.04 A
IZAV		3.44 ± 0.39 B	6.20 ± 0.59 A	16.03 ± 2.39 B	0.94 ± 0.06 A	0.27 ± 0.10 B	4.35 ± 0.65 B	2.12 ± 0.58 B	0.27 ± 0.03 B

^1^ Data are presented as mean ± standard deviation (S.D.) (n = 3). ^2^ Significant differences are evaluated using the Duncan multiple comparison test (small letter) and Student’s *t*-test (capital letter). The different small letters or capital letters in the same column indicate significant differences (*p* < 0.05).

**Table 2:** Organic acid contents of TZAV and IZAV samples ^1,2,3^.

Samples	Aging Time (Year)	Acetic Acid	Lactic Acid	Pyroglutamic Acid	Succinic Acid	Malic Acid	Oxalic Acid	Tartaric Acid
TZAV-1	0	4.22 ± 0.17 cd	1.55 ± 0.03 j	0.82 ± 0.03 g	0.51 ± 0.02 de	0.08 ± 0.01 g	0.11 ± 0.00 e	0.05 ± 0.00 d
TZAV-2	2	4.72 ± 0.15 ab	2.44 ± 0.13 hi	1.43 ± 0.05 d	0.64 ± 0.01 c	0.09 ± 0.01 g	0.14 ± 0.01 cde	0.06 ± 0.01 cd
TZAV-3	5	4.89 ± 0.22 a	2.93 ± 0.06 d	2.26 ± 0.17 a	1.16 ± 0.05 b	0.34 ± 0.01 a	0.21 ± 0.00 a	0.12 ± 0.01 a
TZAV-4	7	4.92 ± 0.17 a	2.72 ± 0.03 ef	2.05 ± 0.11 c	1.37 ± 0.17 a	0.30 ± 0.01 b	0.20 ± 0.00 ab	0.11 ± 0.01 ab
IZAV-1	0	1.58 ± 0.08 e	1.16 ± 0.05 k	0.22 ± 0.01 h	ND ^4^	0.01 ± 0.00 h	0.01 ± 0.00 f	ND
IZAV-2	1	3.92 ± 0.10 cd	2.77 ± 0.09 ef	1.33 ± 0.05 de	0.17 ± 0.00 g	0.13 ± 0.01 f	0.23 ± 0.01 a	0.05 ± 0.01 d
IZAV-3	2	3.86 ± 0.15 d	3.11 ± 0.08 c	1.48 ± 0.08 d	0.54 ± 0.04 d	0.22 ± 0.03 d	0.17 ± 0.01 bc	0.07 ± 0.03 bcd
IZAV-4	3	4.45 ± 0.21 bc	2.56 ± 0.10 gh	1.45 ± 0.07 d	0.09 ± 0.02 g	0.27 ± 0.02 bc	0.13 ± 0.01 de	0.10 ± 0.03 abc
IZAV-5	4	4.03 ± 0.09 cd	3.41 ± 0.06 a	1.64 ± 0.05 c	0.29 ± 0.01 f	0.18 ± 0.01 e	0.16 ± 0.01 cd	0.06 ± 0.01 cd
IZAV-6	4.5	4.56 ± 0.16 b	2.84 ± 0.05 de	1.23 ± 0.04 ef	0.30 ± 0.00 f	0.23 ± 0.04 d	0.12 ± 0.02 e	0.08 ± 0.01 abcd
IZAV-7	5	4.09 ± 0.33 cd	3.27 ± 0.09 b	1.18 ± 0.04 f	0.28 ± 0.00 f	0.13 ± 0.03 f	0.14 ± 0.05 cde	0.10 ± 0.05 abc
IZAV-8	6	4.65 ± 0.11 ab	2.37 ± 0.11 i	1.75 ± 0.10 c	0.33 ± 0.01 f	0.23 ± 0.01 d	0.17 ± 0.03 bc	0.10 ± 0.02 abc
IZAV-9	7	4.12 ± 0.12 cd	2.65 ± 0.06 fg	2.13 ± 0.14 ab	0.44 ± 0.00 e	0.24 ± 0.01 cd	0.13 ± 0.01 de	0.09 ± 0.00 abcd
Mean values								
TZAV		4.68 ± 0.33 A	2.41 ± 0.55 A	1.64 ± 0.60 A	0.92 ± 0.38 A	0.20 ± 0.12 A	0.17 ± 0.04 A	0.09 ± 0.03 A
IZAV		3.91 ± 0.90 B	2.68 ± 0.64 A	1.37 ± 0.51 A	0.31 ± 0.14 B	0.18 ± 0.08 A	0.14 ± 0.06 A	0.08 ± 0.03 A

^1^ Data are presented as mean ± S.D. (n = 3). ^2^ Significant differences are evaluated using the Duncan multiple comparison test (small letter) and Student’s *t*-test (capital letter). The different small letters or capital letters in the same column indicate significant differences (*p* < 0.05). ^3^ Organic acid contents expressed as g/100 mL. ^4^ ND indicates not detected.

**Table 3:** Total phenolic content (TPC), total flavonoid content (TFC) and total antioxidant activity (TAA) (measured by 2,2-diphenyl-1-picrylhydrazyl (DPPH), ferric reducing antioxidant power (FRAP) and 2,2′-azino-bis(3-ethylbenzthiazoline-6-sulfonic acid) (ABTS) assays) in TZAV and IZAV ^1,2^.

Samples	Aging Time (Year)	TPC (mg GAE/mL)	TFC (mg RE/mL)	TAC (mmol TEAC/L)		
				DPPH	FRAP	ABTS
TZAV-1	0	2.49 ± 0.03 g	1.57 ± 0.03 f	33.32 ± 1.94 cde	11.31 ± 0.48 g	14.29 ± 0.98 e
TZAV-2	2	2.82 ± 0.16 f	1.64 ± 0.11 ef	32.35 ± 1.35 cde	9.83 ± 0.35 h	11.96 ± 0.77 fg
TZAV-3	5	6.45 ± 0.19 a	5.44 ± 0.09 a	54.72 ± 0.79 a	30.85 ± 0.14 a	41.07 ± 1.06 a
TZAV-4	7	5.55 ± 0.19 b	4.15 ± 0.09 b	43.76 ± 1.90 abc	24.03 ± 0.33 b	37.49 ± 1.28 b
IZAV-1	0	2.07 ± 0.01 h	1.21 ± 0.05 g	17.82 ± 1.69 f	9.25 ± 0.77 h	10.47 ± 1.02 g
IZAV-2	1	2.58 ± 0.17 fg	2.23 ± 0.03 d	26.06 ± 0.79 ef	13.44 ± 0.88 f	10.74 ± 1.04 g
IZAV-3	2	2.65 ± 0.06 fg	1.88 ± 0.05 e	28.10 ± 2.08 def	13.41 ± 1.25 f	11.85 ± 2.28 fg
IZAV-4	3	3.34 ± 0.06 e	2.26 ± 0.09 d	37.08 ± 2.24 cde	13.72 ± 0.35 f	13.93 ± 0.67 ef
IZAV-5	4	3.34 ± 0.07 e	2.40 ± 0.08 d	38.99 ± 0.79 cd	13.97 ± 0.47 f	17.28 ± 0.18 d
IZAV-6	4.5	3.42 ± 0.19 e	2.85 ± 0.09 c	43.39 ± 1.91 abc	19.80 ± 0.19 c	20.74 ± 1.82 e
IZAV-7	5	3.35 ± 0.09 e	2.38 ± 0.04 d	44.51 ± 0.81 abc	20.79 ± 0.56 c	21.25 ± 1.49 c
IZAV-8	6	4.07 ± 0.11 c	3.00 ± 0.07 c	53.01 ± 1.81 ab	15.54 ± 0.39 e	21.52 ± 0.65 c
IZAV-9	7	3.76 ± 0.14 d	2.77 ± 0.16 c	40.65 ± 1.14 bcd	16.65 ± 0.75 d	19.35 ± 1.19 cd
Mean values						
TZAV		4.33 ± 1.79 A	3.20 ± 1.73 A	41.04 ± 12.45 A	19.01 ± 9.18 A	26.20 ± 13.78 A
IZAV		3.16 ± 0.61 B	2.37 ± 0.60 A	36.62 ± 11.18 A	15.18 ± 3.45 A	16.34± 4.60 B

^1^ Data are presented as mean ± S.D. (n = 3). ^2^ Significant differences are evaluated using the Duncan multiple comparison test (small letter) and Student’s *t*-test (capital letter). The different small letters or capital letters in the same column indicate significant differences (*p* < 0.05).

**Table 4:** Correlations between TAA of TZAV and IZAV samples and their TPC and TFC ^1,2^.

	DPPH		FRAP		ABTS	
	TZAV	IZAV	TZAV	IZAV	TZAV	IZAV
**TPC**	0.963 *	0.960 **	0.988 *	0.682 *	0.987 *	0.872 *
**TFC**	0.985 *	0.903 **	0.997 *	0.724 *	0.981 *	0.824 *

^1^ Correlations are evaluated using Pearson’s correlation test. * and ** indicate the significant levels at 0.05 and 0.01, respectively. ^2^ Different numbers represent Pearson correlation coefficient (−1 ≤ r ≤ 1).

**Table 5:** Compositions of phenolic compound in TZAV and IZAV samples ^1,2,3^.

Samples	Aging Time (Year)	P-hydroxybenzoic Acid	Chlorogenic Acid	Caffeic Acid	Vanillic Acid	Syringic Acid	Catechin	P-coumaric Acid	Ferulic Acid	Sinapic Acid	Rutin
TZAV-1	0	7.96 ± 1.05 b	2.77 ± 0.38 fg	4.46 ± 1.45 cd	4.47 ± 0.97 d	2.32 ± 0.18 ef	44.66 ± 2.68 hi	1.01 ± 0.09 a	0.89 ± 0.06 h	2.66 ± 0.30 def	9.88 ± 0.98 c
TZAV-2	2	4.47 ± 0.27 ef	3.75 ± 0.00 fg	5.57 ± 0.96 abcd	5.78 ± 0.82 cd	2.08 ± 0.31 efg	47.09 ±1.42 gh	1.11 ± 0.01 a	1.30 ± 0.08 h	2.11 ±0.25 ef	10.11 ±1.58 c
TZAV-3	5	10.90 ± 0.02 a	15.09 ± 0.01 b	7.37 ± 0.14 a	5.82 ± 0.06 cd	24.39 ± 0.01 a	39.86 ± 0.09 i	0.35 ± 0.00 b	6.41 ± 0.00 b	5.24 ± 0.03 ab	18.50 ± 0.31 a
TZAV-4	7	6.52 ± 0.04 f bc	6.23 ± 0.07 d	3.66 ± 0.03 d	6.97 ± 0.02 c	22.46 ± 0.01 b	33.81 ± 1.16 j	0.19 ± 0.04 c	17.98 ± 0.05 a	4.43 ± 0.07 bc	14.03 ± 0.19 b
IZAV-1	0	6.22 ± 2.15 cd	4.32 ± 0.05 ef	1.61 ± 0.21 e	7.75 ± 0.09 bc	2.83 ± 1.22 ef	81.12 ± 1.26 a	ND ^4^	5.41 ± 0.23 c	3.81 ± 1.36 bcd	ND
IZAV-2	1	6.20 ± 0.24 cd	6.49 ± 1.75 d	5.57 ± 0.36 abcd	6.28 ± 0.25 cd	2.69 ± 0.24 ef	62.28 ± 1.43 de	ND	2.91 ± 0.24 de	6.54 ± 0.26 a	ND
IZAV-3	2	4.89 ± 0.13 def	3.29 ± 0.07 fg	5.08 ± 0.15 bcd	4.40 ± 0.21 d	3.00 ± 0.07 e	67.49 ± 0.23 bc	ND	4.90 ± 0.02 c	2.83 ± 0.09 cdef	ND
IZAV-4	3	2.83 ± 0.09 g	6.20 ± 0.15 d	5.13 ± 1.79 bcd	5.90 ± 2.43 cd	2.76 ± 0.58 ef	57.80 ± 0.84 ef	ND	2.01 ± 0.15 g	1.88 ± 0.10 ef	ND
IZAV-5	4	4.10 ± 0.25 efg	8.23 ± 1.50 c	6.04 ± 0.15 abc	9.10 ± 0.26 ab	5.38 ± 0.48 c	63.37 ± 6.87 cd	ND	2.69 ± 0.58 ef	3.09 ± 1.74 cde	ND
IZAV-6	4.5	2.70 ± 0.10 g	2.14 ± 0.25 g	5.01 ± 0.59 bcd	9.03 ± 0.90 ab	4.00 ± 0.00 d	57.06 ± 0.46 f	ND	3.35 ± 0.08 d	1.45 ± 0.04 ef	ND
IZAV-7	5	1.22 ± 0.05 h	2.50 ± 0.03 g	6.45 ± 0.58 abc	6.24 ± 1.10 cd	4.02 ± 0.05 d	70.20 ± 0.09 b	ND	1.90 ± 0.00 g	1.59 ± 0.02 ef	ND
IZAV-8	6	3.65 ± 0.28 fg	5.68 ± 0.26 de	5.48 ± 1.72 abcd	9.59 ± 0.49 ab	1.89 ± 0.38 fg	65.30 ± 0.86 bcd	ND	2.26 ± 0.47 fg	1.29 ± 0.06 f	ND
IZAV-9	7	5.54 ± 0.01 cde	20.00 ± 0.75 a	6.81 ± 0.12 ab	10.31 ± 0.00 a	1.17 ± 0.33 g	51.87 ± 1.20 g	ND	0.96 ± 0.10 h	2.84± 0.99 cdef	ND
Mean values											
TZAV		7.46 ± 2.54 A	6.96 ± 5.19 A	5.27 ± 1.63 A	5.76 ± 1.06 B	12.81 ± 11.37 A	41.35 ± 5.56 B	0.66 ± 0.43	6.65 ± 7.37 A	3.61 ± 1.37 A	13.13 ± 3.82
IZAV		4.15 ± 1.75 B	6.54 ± 5.30 A	5.24 ± 1.59 A	7.62 ± 2.07 A	3.08 ± 1.27 B	64.17± 8.71 A		2.83 ± 1.52 A	2.81 ± 1.70 A	

^1^ Data are presented as mean ± S.D. (n = 3). ^2^ Significant differences are evaluated using the Duncan multiple comparison test (small letter) and Student’s *t*-test (capital letter). The different small letters or capital letters in the same column indicates significant difference (*p* < 0.05). ^3^ Phenolic compounds contents expressed as mg/L. ^4^ ND indicates not detected.

**Table 6:** Correlations between TAA of TZAV and IZAV samples and their main phenolic compounds contents ^1,2^.

Phenolic Compounds	DPPH		FRAP		ABTS	
	TZAV	IZAV	TZAV	IZAV	TZAV	IZAV
**p-hydroxybenzoic acid**	0.784	−0.693 *	0.725	−0.749 *	0.626	−0.674 *
**Chlorogenic acid**	0.960 *	0.111	0.903	−0.039	0.814	0.135
**Caffeic acid**	0.554	0.664	0.414	0.673 *	0.251	0.577
**Vanillic acid**	0.432	0.481	0.532	0.201	0.636	0.596
**Syringic acid**	0.929 *	0.012	0.976*	0.178	0.998 **	0.080
**Catechin**	−0.631	−0.492	−0.749	−0.454	−0.855	−0.386
**p-coumaric acid**	−0.834	-	−0.913	-	−0.971 *	-
**Ferulic acid**	0.475	−0.711 *	0.610	−0.578	0.742	−0.636
**Sinapic acid**	0.971 *	−0.717 *	0.994 **	−0.540	0.987 *	−0.725 *
**Rutin**	0.998 **	-	0.979 *	-	0.929 *	-

^1^ Correlations are evaluated using the Pearson’s correlation test. * and ** indicate the significant levels at 0.05 and 0.01, respectively. ^2^ Different numbers represent Pearson correlation coefficient (−1 ≤ r ≤ 1).

## Supplementary Materials

The following are available online, Table S1: Statistical evaluation of the calibration data of organic acids contents by the HPLC method, Table S2: Statistical evaluation of the calibration data of phenolic compound content by the HPLC method.

## Abbreviations

ABTS	2,2′-azino-bis(3-ethylbenzthiazoline-6-sulfonic acid
ANOVA	analysis of variance
DPPH	2,2-diphenyl-1-picrylhydrazyl
FRAP	ferric reducing antioxidant power
GAE	gallic acids equivalents
HPLC	high performance liquid chromatography
IZAV	industrial Zhenjiang aromatic vinegar
LOD	limits of detection
LOQ	limits of detection quantitation
PC	principal components
PCA	principal component analysis
RE	rutin equivalents
TAA	total antioxidant activity
TEAC	Trolox equivalent antioxidant capacity
TFC	total flavonoid content
TPC	total phenolic content
TEAC	Trolox equivalent antioxidant capacity
TZAV	traditional Zhenjiang aromatic vinegar
ZAV	Zhenjiang aromatic vinegar
