# Supplementary material for: Chemical Composition and Antioxidant Characteristic of Traditional and Industrial Zhenjiang Aromatic Vinegars during the Aging Process
Source: Molecules. 2018 Nov 12;23(11):2949. doi: 10.3390/molecules23112949 (PMC6278357; doi:10.3390/molecules23112949)
Supplement: Supplementary file 1 [file molecules-23-02949-s001.pdf]

Supplementary Materials

Table S1. Statistical evaluation of the calibration data of organic acids contents by HPLC method.

**Organic acids**	**Retention time**	**Slope**	**Intercept**	**Correl coeff**	**LOD (μg/mL)**	**LOQ (μg/mL)**
Oxalic acid	7.789	92409	1007.7	0.9998	0.01	0.03
Tartaric acid	9.904	74357	307.69	0.9994	0.01	0.03
Malic acid	10.868	23429	72.75	0.9996	0.03	0.09
Succinic acid	13.168	46177	560.63	0.9996	0.02	0.04
Lactic acid	14.325	6593.5	105.26	0.9998	0.02	0.06
Acetic acid	16.082	7849.9	157.67	0.9998	0.02	0.08
Pyroglutamic acid	20.661	7295.9	112.68	0.9999	0.03	0.12

Table S2. Statistical evaluation of the calibration data of phenolic compounds contents by HPLC method.

**Phenolic compounds**	**Retention time**	**Slope**	**Intercept**	**Correl coeff**	**LOD (μg/mL)**	**LOQ (μg/mL)**
P-hydroxybenzoic acid	15.729	30.029	0.6524	0.9993	0.02	0.06
Chlorogenic acid	18.981	20.9	136.57	0.9998	0.03	0.12
Caffeic acid	20.493	53.11	12.266	0.9992	0.02	0.06
Vanillic acid	21.576	34.647	24.074	0.9997	0.02	0.08
Syringic acid	24.565	49.395	45.142	0.9992	0.01	0.04
Catechin	27.942	7.782	228.38	0.9998	0.05	0.20
P-coumaric acid	29.816	89.411	67.915	0.9998	0.02	0.10
Ferulic acid	35.109	54.847	0.1822	0.9999	0.02	0.06
Sinapic acid	36.102	17.375	41.623	0.9996	0.03	0.12
Rutin	38.167	9.8001	31.168	0.9998	0.04	0.12
